# Chassis organism from *Corynebacterium glutamicum* – a top-down approach to identify and delete irrelevant gene clusters

**DOI:** 10.1002/biot.201400041

**Published:** 2014-10-08

**Authors:** Simon Unthan, Meike Baumgart, Andreas Radek, Marius Herbst, Daniel Siebert, Natalie Brühl, Anna Bartsch, Michael Bott, Wolfgang Wiechert, Kay Marin, Stephan Hans, Reinhard Krämer, Gerd Seibold, Julia Frunzke, Jörn Kalinowski, Christian Rückert, Volker F Wendisch, Stephan Noack

**Affiliations:** 1 Institute of Bio- and Geosciences, IBG-1: Biotechnology, Systems BiotechnologyForschungszentrum Jülich, Jülich, Germany; 2 Institute of Bio- and Geosciences, IBG-1: Biotechnology, Systemic MicrobiologyForschungszentrum Jülich, Jülich, Germany; 3 Chair of Genetics of Prokaryotes, Faculty of Biology & CeBiTec, Bielefeld UniversityBielefeld, Germany; 4 Institute of Biochemistry, University of CologneCologne, Germany; 5 Evonik Degussa GmbHHalle/Westphalia, Germany; 6 Microbial Genomics and Biotechnology, Center for Biotechnology, Bielefeld UniversityBielefeld, Germany

**Keywords:** *Corynebacterium glutamicum*, Chassis, Growth studies, Microbial phenotyping, Relevant genes

## Abstract

For synthetic biology applications, a robust structural basis is required, which can be constructed either from scratch or in a top-down approach starting from any existing organism. In this study, we initiated the top-down construction of a chassis organism from *Corynebacterium glutamicum* ATCC 13032, aiming for the relevant gene set to maintain its fast growth on defined medium. We evaluated each native gene for its essentiality considering expression levels, phylogenetic conservation, and knockout data. Based on this classification, we determined 41 gene clusters ranging from 3.7 to 49.7 kbp as target sites for deletion. 36 deletions were successful and 10 genome-reduced strains showed impaired growth rates, indicating that genes were hit, which are relevant to maintain biological fitness at wild-type level. In contrast, 26 deleted clusters were found to include exclusively irrelevant genes for growth on defined medium. A combinatory deletion of all irrelevant gene clusters would, in a prophage-free strain, decrease the size of the native genome by about 722 kbp (22%) to 2561 kbp. Finally, five combinatory deletions of irrelevant gene clusters were investigated. The study introduces the novel concept of relevant genes and demonstrates general strategies to construct a chassis suitable for biotechnological application.

See accompanying commentary by Víctor de Lorenzo DOI 10.1002/biot.201400493

## 1 Introduction

Synthetic biology aims to introduce engineering principles into the life sciences in order to allow the rational design of biological devices from scratch. Two main prerequisites are a library of well-characterized genetic entities and a robust structural basis [1]. The latter is regularly referred to as a minimal organism and described as a cell with the essential properties of any living organ-ism, such as: (i) encapsulation, (ii) storage of information, (iii) gene expression, and (iv) cell replication. For synthetic biology applications, such a minimal organism should not influence the function of any inserted genetic device (orthogonality principle) and should display a minimal biological complexity in order to ensure full predictability of the behavior of the constructed system [2].

The concept of a minimal organism is quite appealing and thus several attempts to construct biological systems with minimized gene sets have been undertaken in the past few years [3, 4]. The results obtained in these studies, however, were often not directly comparable to each other, as the target criteria were unequal or not properly defined. The importance of formulating clear criteria, keeping applications in mind, and precisely defining the termini used for synthetic biology projects has also been emphasized in a recent critical commentary [5].

**Figure 1:**
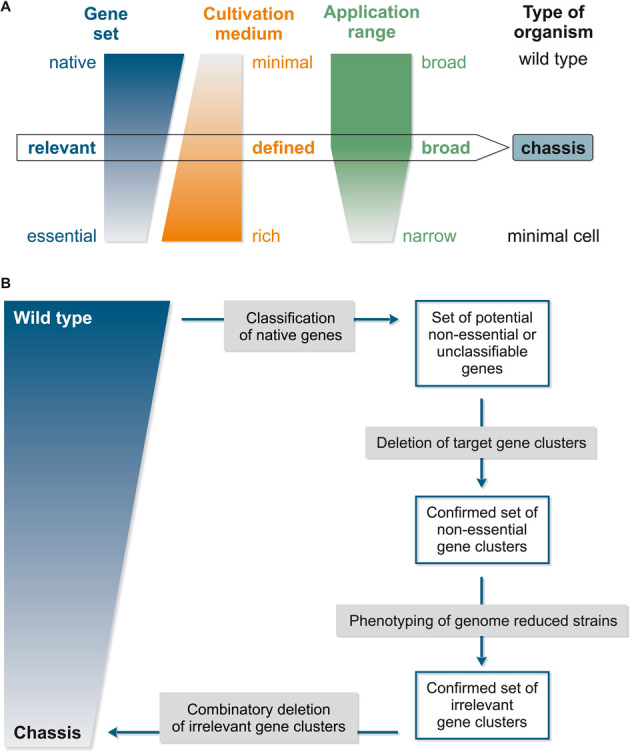
Definitions and workflow for the construction of a chassis organism of *Corynebacterium glutamicum*. (A) Definitions considering the interplay of gene set, cultivation medium, and application range for different types of organisms. (B) Scheme of our targeted top-down approach toward a chassis covering only genes that are relevant for growth on defined medium and maintaining the broad application range of the wild-type organism.

We define a minimal cell as an organism that is restricted to its essential gene set and can grow exclusively in a highly enriched growth medium (Fig. 1A). In contrast, a chassis is defined as an organism that maintainsas the growth behavior and application range of the respective wild type. Consequently, the genome of a chassis is larger than that of a minimal cell, since certain gene functions must be present in addition to the essential gene set. This henceforth called “relevant” gene set should ensure the biological fitness of the chassis at wild-type level under any predefined condition (e.g. exponential growth on defined medium). By definition, a relevant gene set cannot be minimal as it still covers a fully functional anabolism, which is required to establish a reasonable host for a broad range of biotechnological applications.

A chassis construction can be conducted either following a top-down or a bottom-up strategy. The latter would include the synthesis and linkage of all cell components, starting from an artificial membrane system and ending at a synthetic chromosome. Such a rigorous engineering approach is, at the moment, still too complicated and not yet ready for routine application, although the transfer of a single chromosome between two mycoplasma species was reported recently [6]. Hence, the currently feasible and promising way to construct a chassis is the top-down strategy, in which an existing cell is trimmed to its relevant genome following a targeted or untargeted approach. A prerequisite for the latter is a tool that excises parts of the starting genome in a completely randomized distribution without of hot-spots and which allows a subsequent annotation of the deletion sites. An advantage of this method is that it does not require prior detailed knowledge of the starting organism. However, if such pre-knowledge is available, the chassis can be constructed by deletion of specific fragments, which are considered “irrelevant” in a targeted top-down approach (Fig. 1B).

In our collaborative project, we construct a chassis based on the gram-positive soil bacterium *Corynebacterium glutamicum* ATCC 13032 [7]. Since its isolation due to its natural l-glutamate excretion capacity, the product spectrum of *C. glutamicum* has been broadened to different chemicals, materials, and fuels in multiple metabolic engineering approaches over the last few decades [8]. Following these approaches, a deep insight was gained into the different omics layers of *C. glutamicum* (e.g. see [9–12]), with the first highlight being complete genome sequencing in 2003 [13]. This existing knowledge together with potential industrial applications makes *C. glutamicum* a promising target for the construction of a chassis. In a previous publication, the targeted deletion of 11 distinct regions with a total size of 250 kilobase pair (kbp) was reported in the strain R [14]. The most successful trial in terms of deletion size was, however, performed via insertion and excision in an untargeted approach, but resulted in multiple growth defects of the constructed strains [15].

In contrast, we construct a chassis organism following a targeted top-down approach by step-wise reduction of the native genome of *C. glutamicum* based on prior estimations of gene essentiality (cf. Fig. 1B). Recently, we reported on the construction and characterization of the prophage-free strain MB001 [16], which showed an overall genome reduction of about 6% and an increased transformation efficiency and plasmid copy number. On this basis, we continued our work and classified the native genes of *C. glutamicum* with respect to their essentiality on a widely used, defined medium, namely CGXII mineral salts medium with D-glucose [17]. In the next step, we deleted several clusters of non-essential as well as unclassifiable genes from the prophage-free strain and evaluated the growth phenotype of the truncated strains on CGXII medium. This phenotyping step is crucial to filter the set of non-essential gene clusters for those that are irrelevant for maintaining the biological fitness of the wild type (WT). In the chosen approach, biological fitness was assessed by the specific growth rate because a drop in this easily measureable parameter directly indicates an altered metabolism of the particular strain. Finally, we identified a set of 26 gene clusters irrelevant to the biological fitness of *C. glutamicum*, and present the first results from the combinatory deletion of multiple gene clusters, paving the way toward a *C. glutamicum* chassis.

## 2 Materials and methods

### 2.1 Identification of essential genes

Genome sequencing was performed on 454 GS-FLX (454, Branford, CT, USA) and MiSeq (Illumina, Chesterford, UK) sequencing platforms using whole genome and 8k paired-end (GS-FLX) or paired-end and 8k MatePair (MiSeq) libraries, respectively. The NGS sequencing data was assembled using Newbler v2.3 and v2.6 (454, Branford, CT, USA) and in silico finishing was performed using the Phred/Phrap/Consed software package [18]. All genomes were annotated using the GenDB annotation pipeline [19] to predict coding regions and perform functional annotation. The annotated genomes were then used to perform core genome analysis with EDGAR [20] from which the gene conservation codes were derived.

To classify genes based on their relative transcript abundance, the recently published whole transcriptome data set for *C. glutamicum* [21] was used. These data include reads of fragmented RNA and were therefore normalized by the number of reads per kilobase gene length and million mapped reads (*RPKM*) to obtain a comparable value for the relative transcription of each gene.

### 2.2 Deletion of gene clusters

All oligonucleotides used for plasmid construction and testing of the deletion strains are listed in Supporting information, Table S1. Gene clusters were deleted from the *C. glutamicum* genome by double crossover as described previously for single genes [22]. In short, *C. glutamicum* was first transformed by electroporation with the sequenced deletion plasmids. These are derivatives of the suicide plasmid pK19*mobsacB* that cannot replicate in *C. glutamicum.* Therefore, all kanamycin-resistant clones should have integrated the plasmid into the chromosome by homologous recombination at one of the flanks of the deletion site. To screen for a second recombination event, recombinants were cultivated without kanamycin for 6 h and subsequently plated onto brain heart infusion supplemented (BHIS) agar plates containing 10% (w v^–1^) sucrose. The *sacB* gene on the plasmid encodes levansucrase, an enzyme that is lethal to *C. glutamicum* in the presence of sucrose. Therefore, all colonies appearing on the sucrose plates should have lost the plasmid by a second homologous recombination, which either restores the wild-type situation or leads to the desired mutation. Kanamycin-sensitive and sucrose-resistant clones were subsequently tested by colony-PCR analysis with the oligonucleotide-pair Dfw/Drv for the respective genome region. Deletions were carried out in the recently reported prophage-free strain MB001 [16] with additional deletion of two insertion elements (ISCg1 and ISCg2) and are denoted as genome-reduced strains (GRS). In addition, selected regions were deleted from the prophage-free L-lysine producer DM1933 to obtain genome-reduced L-lysine producers (GRLP). In total, 60 strains were constructed in this work (Table 1).

**Table 1:** Strains used in this work with deletions indicated by cg numbers.

Deleted gene cluster	Wild-type-based ΔCGP123, ΔISCg12	DM1933-based ΔCGP123
Δ0116-0147	GRS12	–
Δ0158-0183	GRS13	–
Δ0311-0333	GRS15	–
Δ0414-0440	GRS16	GRLP16
Δ0635-0646	GRS17	GRLP17
Δ0704-0748	GRS18	–
Δ0822-0845	GRS19	–
Δ0900-0909	GRS20	GRLP20
ΔrrnB-0931	GRS21	–
Δ1018-1033	GRS22	–
Δ1172-1213	GRS23	GRLP23
Δ1219-1247	GRS24	–
Δ1281-1289	GRS25	–
Δ1291-1305	GRS26	–
Δ1340-1353	GRS28	–
Δ1370-1385	GRS29	–
Δ1540-1549	GRS30	GRLP30
Δ1843-1853	GRS31	GRLP31
Δ2136-2139	GRS32	GRLP32
Δ2312-2322	GRS33	–
Δ2621-2643	GRS37	GRLP37
Δ2663-2686	GRS38	–
Δ2701-2716	GRS39	GRLP39
Δ2755-2760	GRS40	GRLP40
Δ2801-2828	GRS41	GRLP41
Δ2880-2904	GRS42	GRLP42
Δ2925-2943	GRS43	GRLP43
Δ2965-2973	GRS44	–
Δ2990-3006	GRS45	GRLP45
Δ3050-3062	GRS46	GRLP46
Δ3072-3091	GRS47	GRLP47
Δ3102-3111	GRS48	GRLP48
Δ3208-3236	GRS50	GRLP50
ΔrrnC-3298	GRS51	–
Δ3324-3345	GRS53	GRLP53
Δ3365-3413	GRS54	–
**Combined cluster deletions**		
Δ1172-1213 Δ0414-0440	GRS16_23	–
Δ1172-1213 Δ1018-1033	GRS22_23	
Δ1172-1213 Δ3050-3062	GRS23_46	–
Δ2801-2828 ΔrrnB-0931	GRS21_41	–
Δ2801-2828 ΔrrnC-3298	GRS41_51	–

### 2.3 Growth medium

All chemicals used for growth media were purchased from Sigma–Aldrich. Cultivations were performed either on defined or enriched CGXII medium. Both media variants contained per liter of distilled water: 1 g K_2_HPO_4_, 1 g KH_2_PO_4_, 5 g urea, 13.25 mg CaCl_2_ · 2H_2_O, 0.25 g MgSO_4_ · 7H_2_O, 1 mg FeSO_4_ · 7H_2_O, 1 mg MnSO_4_ · H_2_O, 0.02 mg NiCl_2_ · 6H_2_O, 0.313 mg CuSO_4_ · 5H_2_O, 1 mg ZnSO_4_ · 7H_2_O, 30 mg protocatechuic acid (PCA), 42 g 3-(*N*-morpholino)propanesulfonic acid (MOPS) buffer, and 10 g or 40 g D-glucose. The concentration of (NH_4_)_2_SO_4_ was 20 or 10 g L^–1^ and biotin was added to 0.2 or 0.25 mg L^–1^ in defined or enriched CGXII, respectively. Enriched CGXII was furthermore supplemented per liter of distilled water with: 6 g yeast extract, 238 mg L-threonine, 0.5 mg thiamine–HCl, 0.1 mg cyanocobalamin, and 2.5 mg pyridoxine–HCl. During medium preparation, 4 M NaOH was used to adjust pH 7.0 and some substances were added sterile after autoclaving (D-glucose, PCA, biotin, trace elements, and vitamins).

### 2.4 Strain storage and cultivation

Strains with successfully deleted gene clusters were grown on LB or CASO agar plates and colonies were suspended in 0.9% (w v^–1^) NaCl with 20% (v v^–1^) glycerol and stored at –80°C as a master cell bank (MCB). To generate working cell banks (WCBs), 10 μL of each MCB was added to 990 μL medium with 40 g L^–1^ D-glucose in a Flowerplate® (m^2^p-labs, Baesweiler, Germany) and incubated for 48 h in a shaking device at 1000 revolutions per minute (rpm), 75% humidity, and 30°C. Each strain was grown separately on defined as well as on enriched CGXII medium. Subsequently, the optical density (OD_600_) in each well was measured and adjusted to OD_600_ = 20 with 0.9% (w v^–1^) NaCl. Finally, glycerol was added to 20% (v v^–1^) and the derived WCB was stored in aliquots in sterile microtiter plates (MTP) at –80°C.

The main cultures were started at an OD_600_ of 0.2 by inoculating 990 μL medium with 10 μL of a WCB, which had already been grown on the same medium (defined or enriched CGXII). Each WCB-MTP was used only once and discarded after use. Reference strains (WT or DM1933) were cultivated on every plate as a control. Growth experiments were performed in Flowerplates with pH and pO_2_ optodes in a BioLector (m^2^p-labs) at 1000 rpm, 95% humidity, 30°C, and backscatter (BS) gain 20. For subsequent data analysis, each BS curve was first blanked by its initial value. Secondly, all blanked values below the limit of quantification (BS value: 10) were deleted from the data set. Finally, maximum specific growth rates were determined by fitting exponential functions to the BS data of corresponding exponential growth phases, while the end of exponential growth was determined by the time when pO_2_ stopped dropping in each cultivation. The obtained growth rates for all strains were analyzed for significant changes compared to reference organisms (WT or DM1933). First, the *f*-test (*p*<0.01) was used to check if the variance of the growth rate distribution of the particular strain and the reference showed significant alterations. Subsequently, the two-sample *t*-test (*p*<0.01) was performed as either a homoscedastic or heteroscedastic test, depending on the outcome of the *f*-test.

## 3 Results

### 3.1 Blueprint of a *C. glutamicum* chassis organism

**Figure 2:**
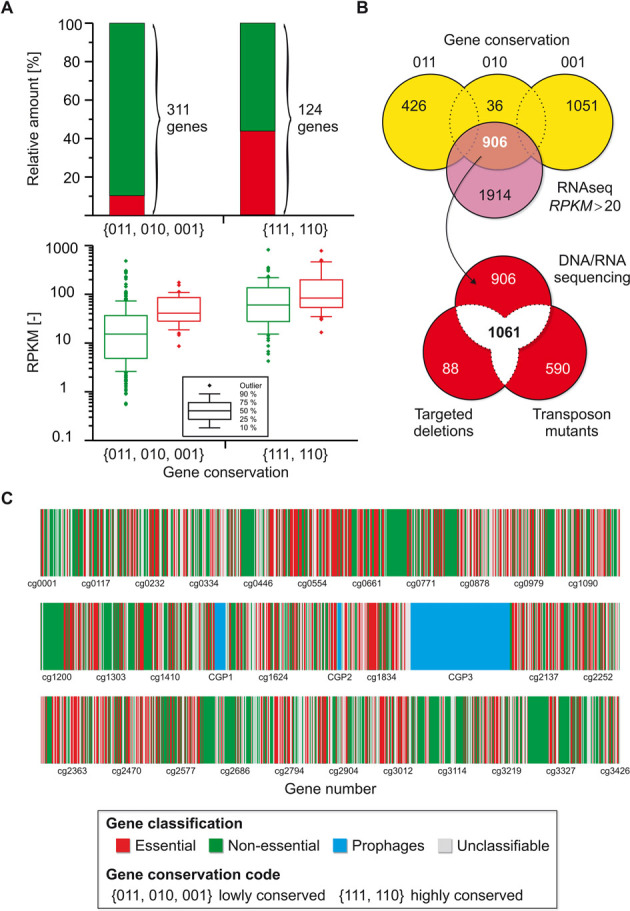
Classification of gene essentiality for *C. glutamicum* ATCC 13032. (A) A priori analysis to determine potential correlations in the expression and conservation of genes based on an initial subset of 435 genes. (B) VENN diagrams from the identification of essential genes combining data from genome and RNA sequencing as well as targeted and untargeted knockout studies. (C) Genome map with classification results of essential, non-essential, and unclassifiable genes. The three prophages of *C. glutamicum* (CGP123) were already deleted in our previous work [16].

To enable the targeted construction of a chassis, we first established a genome-wide classification of essential genes of *C. glutamicum* ATCC 13032. Therefore, we integrated data from RNA and DNA sequencing together with published as well as unpublished data from all authors (Fig. 2).

In the first step, we investigated the degree of conservation of each gene in the species and genus of *C. glutamicum* and determined three core genomes, which represent different degrees of phylogenetic proximity to ATCC 13032. The core genome in the closest distance was called the species core and included genes that were found in all of the following *C. glutamicum* strains: *C. glutamicum* ATCC 13032 (NC_006958) [13], “*Brevibacterium lactofermentum*” ATCC 13869 (unpublished genome), ”*Brevibacterium flavum*” ATCC 14067 (unpublished genome), “*Corynebacterium* sp.” ATCC 14747 (unpublished genome), “*Micrococcus glutamicum*” ATCC 14752 (unpublished genome), “*Arthrobacter albidus*” ATCC 15243 (unpublished genome), “*C. melassecola*” ATCC 17965 (unpublished genome), “*C. crenatum*” NCC 1.542 (unpublished genome), *C. glutamicum* LP-6 (unpublished genome), and *C. glutamicum* R (NC_009342) [23]. An intermediate degree of phylogenetic proximity was displayed by the subgroup core, which included gene overlaps from genomes of the subgroup within the genus *Corynebacterium*, such as: *C. glutamicum* ATCC 13032 (NC_006958) [13], *C. deserti* (unpublished genome), *C. efficiens* (NC_004369) [24] and *C. callunae* (unpublished genome). Finally, the widest core genome was called the genus core and included genes that were found in all of the following organisms: *C. diphtheria* (NC_002935) [25], *C. halotolerans* (NC_020302) [26], *C. jeikeium* (NC_007164) [27], *C. nuruki* (AFIZ00000000) [28], *C. maris* (NC_021915) [29], *C. resistens* (NC_015673) [30], *C. terpenotabidum* (NC_021663) [31], *C. variabile* (NC_015859) [32], and all genomes from the subgroup core. In order to evaluate the conservation for each gene, the information as to whether a gene belongs to a certain core genome was converted into a three-bit conservation code, with each bit representing a core genome group of the following order: genus, subgroup, and species. Thus, a code of “111” indicates presence in all three core genomes, while a gene with the conservation code “001” is only present in the species core genome.

In a further analysis, we calculated the relative amount of expressed RNA from a recently published whole transcriptome data set for *C. glutamicum* [21]. Therefore, we normalized the quantified RNA fragments as reads per kilobase gene length and million mapped reads (RPKM) to obtain a comparable value for the relative transcription of each gene. Using this RPKM value and conservation code, we analyzed 435 genes from ATCC 13032, which already had been experimentally confirmed as either essential or non-essential by taking literature data and expert knowledge into account (Fig. 2A). From this a priori analysis, general correlations could be deduced, enabling the prediction of essential genes in the remaining set of uncharacterized *C. glutamicum* genes.

As a first result, nearly half (44 %) of all strictly conserved genes (conservation code: 111 or 110) were identified as essential, while less conserved genes (conservation code: 011, 010, or 001) showed a much lower probability of being essential (10%). Thus, a general correlation between gene conservation and gene essentiality exists for *C. glutamicum*. We subsequently compared the relative gene expression levels (RPKM score) of essential and non-essential genes in our pre-defined set of 435 genes, taking gene conservation into account. In the group of strictly conserved genes (111 or 110), we observed a tendency that essential genes were higher expressed than non-essential genes (median RPKM 84 and 60). However, the differences in relative gene expression were insufficient to clearly distinguish essential from non-essential genes in this group. As no individual prediction was possible and nearly half of all strictly conserved genes were found to be essential, each of these genes was consequently declared as “unclassifiable, ” in our chassis blueprint, unless its essentiality was proven by other data.

In contrast, for genes with low conservation (011, 010, or 001), a prediction based on the RPKM value was possible. This group of genes showed a nearly three-fold difference in expression levels between essential and non-essential genes with median RPKM scores of 41 and 14, respectively. Nearly 90% of those low-conserved but essential genes had an RPKM above 20, which was then defined as the threshold RPKM value to predict other essential genes in the uncharacterized group of low-conserved genes. By combining the conservation of these genes with their relative expression, we predicted 906 genes as essential in *C. glutamicum* ATCC 13032 (Fig. 2B).

Moreover, a list of non-essential genes was integrated from a study with random gene deletions in *C. glutamicum* strain R via transposon mutations [33]. From this counter-selective transposon screen, a list of 590 essential genes was derived. Our essentiality analysis was finalized by taking targeted deletions in published and unpublished studies into account, resulting in 88 further essential gene hits (partly overlapping with hits from the other approaches). In this step, we also classified all transporters of CGXII media components as well as exporters for by-products and amino acids as essential for our chassis.

In summary, all classification attempts resulted in a consolidated list of 1061 essential genes, which should not be deleted to construct a chassis. Not all of the remaining genes could be classified as non-essential, for example when genes were strictly conserved (as discussed above) or when findings from the literature were in disagreement. Single gene loci were also unclassifiable when they encoded homologous functions together with other genes, as, for example was true for the six ribosomal RNA (rrn) clusters of *C. glutamicum*. In total, 786 genes from ATCC 13032 were unclassifiable while the remaining set of 1362 genes was classified as non-essential.

In the next step, the essentiality information for all genes was mapped as a genome blueprint to identify large groups of neighboring non-essential genes (Fig. 2C). For our top-down approach, we wanted to reduce the genome not by deleting single non-essential genes, but by deleting regions. Using our blueprint, we therefore identified 41 gene clusters with no essential gene ranging from 3.7 to 49.7 kbp in size. We also selected clusters as targets, which included a few unclassifiable genes in order to empirically determine their essentiality.

During the subsequent deletion experiments, the deletion of few of the determined target gene clusters from *C. glutamicum* failed repeatedly, probably because they contained as yet unknown essential genes. However, 36 clusters were successfully deleted from the prophage-free strain MB001 (ΔCGP123 and ΔISCg12). The resulting genome-reduced strains (GRS) directly confirmed our prior genome-wide essentiality classification. Concurrently, the status of any unclassifiable gene in the deleted clusters could be assigned as non-essential. In addition, selected deletions were also carried out in the background of DM1933 to construct genome-reduced L-lysine producers (GRLP). In the following, all strains were phenotypically characterized to evaluate the relevance of the deleted genes for biological fitness and thereby determine valid deletion targets toward a chassis.

### 3.2 Relevance of non-essential gene clusters in different media compositions and strain backgrounds

**Figure 3:**
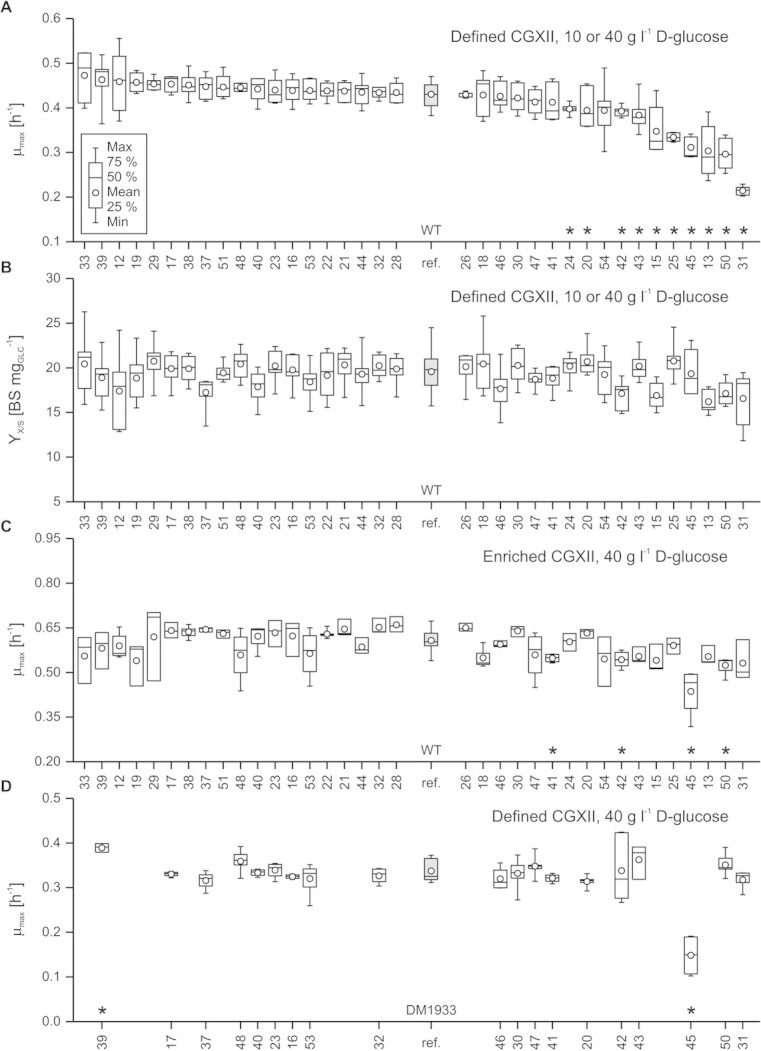
Validation of gene cluster deletions in *C. glutamicum* on the basis of two different strain backgrounds and media compositions. Strains with significant changes in maximum growth rate (*μ*_max_) or biomass yield (*Y*_X/S_) compared to the reference (WT or DM1933) were determined via *t*-test (*p* < 0.01) and are marked by an asterisk. (A) 36 genome-reduced strains (GRS) based on the wild type were cultivated on CGXII medium and sorted according to *μ*_max_ (*n* ≥ 6). (B) Total biomass yields of GRS (*n* ≥ 6). (C) Maximum growth rates of GRS in enriched CGXII medium including L-threonine, vitamins, and yeast extract (*n* ≥ 3). (D) 17 genome-reduced L-lysine producers (GRLP) were obtained by selected gene cluster deletions in the L-lysine producer DM1933 ΔCGP123. The biological fitness of all GRLP was evaluated with regard to *μ*_max_ on CGXII medium and DM1933 was used as a reference (*n* ≥ 3).

In order to test the relevance of all non-essential clusters for a chassis, each GRS was first characterized for changes in biological fitness during growth on defined CGXII medium. Biological fitness was assessed by the maximum specific growth rate (*μ*_max_), since a change in this easily measurable parameter directly indicates an altered metabolism of that particular GRS. The maximum growth rate of each strain was determined from multiple replicates (*n* > 6) of 1 mL batch cultivations in order to generate a valid data set for statistical analysis. Significantly changed *μ*_max_ values were determined via two-sided *t*-tests (*p* < 0.01), comparing each GRS with the *C. glutamicum* WT (Fig. 3A). For the WT, a maximum growth rate of *μ*_max_ = 0.43 ± 0.02 h^–1^ (*n* = 27) was determined, and the rates for the GRS ranged from 0.47 ± 0.06 h^–1^ (GRS33) to 0.22 ± 0.01 h^–1^ (GRS31). In comparison, 10 GRS grew significantly slower, while for 26 GRS the growth rate was maintained at wild-type level.

To further characterize all GRS, the biomass yield was estimated from the maximum backscatter (BS) value and the initial amount of D-glucose (GLC) in each batch culture (Fig. 3B). The biomass yield of the WT was *Y*_X/S_ = 19.6 ± 2.1 BS mg_GLC_^–1^ and only five GRS showed significantly lower yields. Interestingly, these five strains (namely GRS13, GRS15, GRS31, GRS42, and GRS50) also showed a significantly lower *μ*_max_, thereby underlining the relevance of one or multiple genes in the respective non-essential clusters for growth in CGXII medium.

In the next step, biological fitness was tested in a more complex growth medium, which might enable some GRS to compensate potential limitations in the central metabolism created from gene cluster deletions. For this approach, 1 mL cultivations were carried out on CGXII medium enriched with L-threonine, yeast extract, and a vitamin mixture (Fig. 3C). In enriched CGXII medium, a maximum growth rate for the WT of *μ*_max_ = 0.61 ± 0.04 h^–1^ (*n* = 13) was found and the growth rates for the GRS ranged from 0.66 ± 0.03 h^–1^ (GRS28) to 0.44 ± 0.08 h^–1^ (GRS45). Significantly slower growth was observed exclusively for GRS41, GRS42, GRS45, and GRS50. Interestingly, three of these GRS also showed decreased growth rates on defined CGXII medium (cf. Fig. 3A). Conclusively, at least one gene in each of the corresponding deleted gene clusters is of general relevance for the biological fitness of *C. glutamicum* and the addition of complex substrates cannot recover the growth phenotype of the WT. For other GRS, however, the observed impaired biological fitness on CGXII was recovered by adding complex additives and vitamins (i.e. GRS13 and GRS25). Thus, one or more genes in these clusters are relevant for growth on CGXII but are irrelevant on enriched CGXII, indicating a function of the respective genes in anabolic pathways.

To test whether the chosen concept can be transferred to a related strain, a selected set of non-essential gene clusters was also deleted from the prophage-free L-lysine producer DM1933 ΔCGP123. Altogether, 19 GRLP were constructed and compared to the reference strain DM1933 with respect to *μ*_max_ on CGXII medium (Fig. 3D). DM1933 is known to grow slower than the WT [34] and it also showed a lower maximum growth rate of *μ*_max_ = 0.34 ± 0.03 h^–1^ (*n* = 8) in our study. The tested GRLP grew at maximum rates ranging from 0.39 ± 0.01 h^–1^ (GRLP39) to 0.15 ± 0.05 h^–1^ (GRLP45). Most interestingly, both gene clusters (#39 and #45) showed similar consequences for the biological fitness on CGXII medium when deleted in the WT (GRS39 and GRS45) as well as in the DM1933 background (GRLP39 and GRLP45). While GRS39 was ranked second regarding maximum growth rate on CGXII, the analogous GRLP39 grew at the highest rate of all tested GRLP. GRS45 and GRLP45, on the other hand, showed significantly decreased growth performances compared to the reference in all tested conditions. In conclusion, the deletion of selected non-essential gene clusters from DM1933 validated our prior phenotyping results in another strain background.

### 3.3 Theoretical and practical route toward a chassis

**Figure 4:**
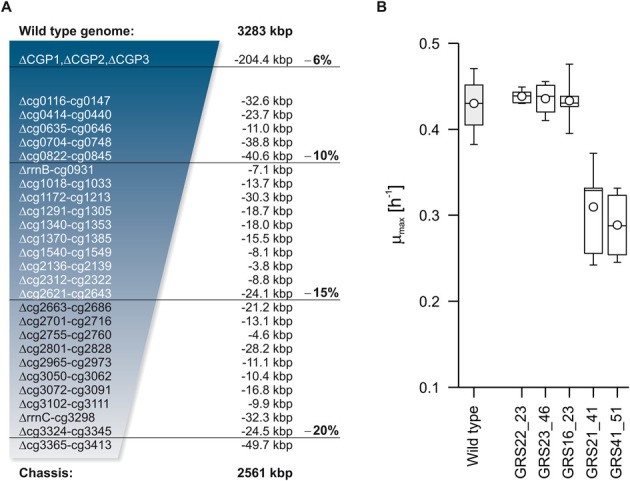
Theoretical and practical combinatory deletion of irrelevant gene clusters to stepwise construct a *C. glutamicum* chassis. (A) Overview of gene clusters that were validated as irrelevant for biological fitness on CGXII medium in this study (cf. Fig. 3). A combined deletion of all of these clusters would theoretically reduce the native genome by about 22% to 2561 kbp. (B) Phenotyping results of second-generation GRS combining double deletions of selected irrelevant gene clusters. Strains were cultivated on CGXII medium with 10 g or 40 g L^–1^ glucose and maximum growth rates were compared to the wild-type reference.

During comprehensive phenotyping of our GRS library, a set of 26 gene clusters was identified to include only such genes, which are irrelevant for the biological fitness of *C. glutamicum* on CGXII medium. Consequently, a combinatorial deletion of all of these clusters in the background of the prophage-free strain MB001 [16] would, theoretically, reduce the native genome size by about 22% from 3283 to 2561 kbp (Fig. 4A).

Such a combinatorial deletion must be carried out sequentially by homologous recombination, as there are currently no techniques available to combine pre-made deletions in *C. glutamicum*. For example, the P1 transduction method, which is frequently applied for *E. coli* [35, 36] is not an option, as there is no functional, well-characterized phage for *C. glutamicum* known to date and *C. glutamicum* is also very resistant to phage induction. During such a sequential deletion procedure, our initial chassis blueprint might alter for a few genes if, for example, potential anti-toxins become non-essential after toxins have been deleted from the genome. Moreover, the simultaneous deletion of multiple clusters is not without risk, especially when relevant functions are encoded redundantly (i.e. isoenzymes) in those clusters.

As a starting point to test the potential of this approach, a small selection of the 26 irrelevant gene clusters was deleted in double combinations, resulting in a handful of second-generation GRS. As a result, two strains (namely GRS21_41 and GRS41_51) displayed a reduced biological fitness when two individually irrelevant gene clusters were deleted simultaneously (Fig. 4B). Thus, cluster #41 (containing cg2801-cg2828, cf. Table 1) was found incompatible with other cluster deletions and should be excluded from further combinations. Nonetheless, the combinatorial strains GRS16_23, GRS22_23, and GRS23_46 grew in a comparable manner to the WT on CGXII medium. These exemplary findings already prove the general applicability of our step-wise top-down approach to construct a *C. glutamicum* chassis.

## 4 Discussion

In the emerging field of synthetic biology, a structural basis is required, which can either be constructed from scratch or in a top-down approach starting from any existing organism. In any case, the identity and quantity of the resulting gene set is greatly influenced by the criteria the organism should fulfill. One of the smallest essential gene sets reported for a minimal cell was found in *Mycoplasma genitalium* and consists of only 485 genes from which 100 can be disrupted one at a time [6]. The growth of *M. genitalium* is, however, comparably slow and requires a highly enriched medium, since this parasitic bacterium lacks many anabolic pathways, for example to synthesize fatty acids or amino acids [37]. Moreover, as an intracellular parasite, it does not require homeostatic mechanisms since a controlled environment is maintained by the host.

In our study, we initiated the construction of a chassis from *C. glutamicum* – an organism which grows fast on defined medium with single carbon sources and is robust against environmental stresses, such as those induced through process inhomogeneity during large-scale cultivation [38]. Therefore, among other things, *C. glutamicum* has been intensively applied for industrial bioprocess development in the past few decades [8]. To maintain the biotechnological potential of *C. glutamicum*, our first criterion for a chassis was its ability to grow on standard CGXII medium with D-glucose as the sole carbon and energy source. Moreover, the growth rate of the chassis should not be negatively affected in comparison to established strains (including wild-type and specific producer mutants), in order to allow for reasonable bioprocesses. In our top down-approach, we first classified all genes from *C. glutamicum* with respect to their essentiality for our target conditions. From this analysis, we identified 41 clusters with a high content of non-essential or unclassifiable genes from which 36 clusters were successfully deleted and thereby all included genes were confirmed as non-essential. On the other hand, new insights into the gene–environment interplay of *C. glutamicum* can also be drawn from clusters where the deletion was repeatedly unsuccessful. These clusters (namely cg0216-cg0232 and cg2348-cg2358) must contain as yet unknown essential gene functions.

From the subsequent phenotypic characterization of all GRS, 26 strains met our criteria of unaltered biological fitness on CGXII medium. Thus, all of these clusters consist exclusively of irrelevant genes and are valid targets for a chassis construction. However, some deletions led to a decreased biological fitness and must therefore include at least one gene of relevant function. Consequently, these clusters should not be considered for chassis construction or must eventually be deleted in a truncated version to retain all relevant genes in the genome. Nevertheless, the observed phenotypes are of high interest, as the results indicate gene functions in the deleted clusters that are not yet fully understood.

One example is GRS25, which grew significantly slower on defined CGXII in comparison to the WT, but showed an equal growth rate on enriched CGXII (cf. Fig. 3A and 3C). One or multiple genes deleted in GRS25 must therefore be relevant on defined CGXII and irrelevant on the enriched medium, indicating that GRS25 is limited in the synthesis of a metabolite, which can be supplemented by enriched CGXII. A reasonable explanation might be the deletion of cg1283 in GRS25, which (in addition to cg1835) is discussed to encode a shikimate dehydrogenase (SDH) for the biosynthesis of aromatic amino acids [13]. In this case, the amino acids from the yeast extract in enriched CGXII would then compensate for the growth defect of GRS25. However, it was recently reported that the single deletion of cg1835 instead of cg1283 led to a more severe growth defect of *C. glutamicum* on defined medium, which is why the main SDH activity was assumed to be encoded by cg1835 [39].

Regarding the interdependence of essential genes and essential medium components, a similar effect was found for GRS13 whose biological fitness was also recovered on enriched CGXII. The deleted cluster includes the gene cg0172 (*panD*), which could not be classified in our essentiality analysis, as unpublished deletion experiments were in disagreement with the literature. However, *panD* encodes an aspartate-1-decarboxylase catalyzing the generation of β-alanine as pantothenate precursor. The limitation observed for GRS13 on defined CGXII might therefore be caused by *panD* deletion but is complemented by components from the yeast extract on enriched CGXII. Interestingly earlier studies on the aspartate-1-decarboxylase reported that the deletion of *panD* from *C. glutamicum* resulted in strictly β-alanine or pantothenate auxotrophic strains [40]. In contrast, GRS13 showed growth on the defined CGXII in all experiments of our study, but at significantly lower rates. Therefore, we conclude that *panD* is not essential for *C. glutamicum*, but probably relevant for its growth on CGXII medium.

An interesting phenomenon was also observed when irrelevant gene clusters were combined in the double deletion strains. Both GRS21_41 and GRS41_51 displayed a reduced biological fitness, although these particular clusters did not affect the growth phenotype when deleted one at a time. A possible reason for this observation could be the homologous functions encoded within the deleted clusters, incorporating one (cluster #21 and #51) or two (cluster #41) operons for ribosomal RNA (rrn). Consequently, the simultaneous deletion of three *rrn* operons in both strains might have limited the ribosome capacity of *C. glutamicum*, which is required for growth rates typically found on CGXII medium. A similar relationship between growth rate and copy number of *rrn* operons was also reported for *E. coli* [41].

A combined deletion of all experimentally confirmed irrelevant clusters from this study would reduce the genome by about 22% (722 kbp) to 2561 kbp in a *C. glutamicum* chassis. However, few deletions might be incompatible during practical combination, as observed for cluster #41. More generally speaking, homologous gene functions must be considered during their combinatory deletion toward a chassis. In comparison, Tsuge et al. [15] reported in 2007 on the untargeted deletion of 42 regions from the *C. glutamicum* strain R with a combined size of 393.6 kbp (11.9%). Here, however, 8 of the 42 strains had lost the ability to grow on minimal media and 23 of the remaining 34 strains grew with a growth rate of less than 90% compared to the WT. The criteria for a chassis set in our study would thereby only have been met by 11 of the 42 strains, which would altogether include 77.4 kbp or 2.3% of the respective strain R.

In prior attempts to rationally trim other industrially relevant organisms to a core production host, the extent of genome reduction ranged between 15.3 and 20.7% for *E. coli* [42] and *B. subtilis* [43], respectively. Interestingly, in multiple recent chassis construction projects, a maximal degree of genome reduction in the range of 15–25% was found repeatedly, independent whether the studied organisms genome was rather small, as was true for *M. genitalium* (580 kbp, [37]) or of a comparably large size, as was the case for *S. avermitilis* (9.02 Mbp, [44]). This observation might indicate a common trend, whereby microorganisms reserve a certain amount of additional genetic information in form of irrelevant genes to gain a higher adaptive capacity to changing environments. The repeatedly achieved maximal genome reduction of 25% might therefore indicate the evolutionary optimal balance between those variable genetic spaces and a core relevant gene set of any bacterium.

## Abbreviations

BS: backscatter
GRLP: genome-reduced L-lysine producer(s)
GRS: genome-reduced strain(s)
kbp: kilobase pair
OD: optical density
RPKM: reads per kilobase gene length and million mapped reads
rrn: ribosomal RNA
WCB: working cell bank
WT: wild type

## Notes

Supporting information available online
