# Supplementary material for: Chassis organism from Corynebacterium glutamicum – a top-down approach to identify and delete irrelevant gene clusters
Source: Biotechnol J. 2014 Oct 8;10(2):290–301. doi: 10.1002/biot.201400041 (PMC4361050; doi:10.1002/biot.201400041)
Supplement: Supplementary file 1 [file biot0010-0290-sd1.pdf]

Biotechnology
Journal

Supporting Information for DOI 10.1002/biot.201400041

Chassis organism from *Corynebacterium glutamicum* – a top-down approach to identify and delete irrelevant gene clusters

Simon Unthan, Meike Baumgart, Andreas Radek, Marius Herbst, Daniel Siebert, Natalie Brühl, Anna Bartsch, Michael Bott, Wolfgang Wiechert, Kay Marin, Stephan Hans, Reinhard Krämer, Gerd Seibold, Julia Frunzke, Jörn Kalinowski, Christian Rückert, Volker F. Wendisch, Stephan Noack

© 2014 Wiley-VCH Verlag GmbH & Co. KGaA, Weinheim

Supplementary Information

Chassis organism from *Corynebacterium glutamicum* – a top-down approach to identify and delete irrelevant gene clusters

Simon Unthan[1], Meike Baumgart[2], Andreas Radek[1], Marius Herbst[3], Daniel Siebert[3], Natalie Brühl[4], Anna Bartsch[4], Michael Bott[2], Wolfgang Wiechert[1], Kay Marin[5], Stephan Hans[5], Reinhard Krämer[4], Gerd Seibold[4], Julia Frunzke[2], Jörn Kalinowski[6], Christian Rückert[6], Volker F. Wendisch[3], Stephan Noack*[1]

Table S1: Primers used in this work to delete gene clusters from *C. glutamicum* via crossover-PCR after Gibson assembly (a), restriction/ligation (b) or blunt-end cloning (c) of the particular deletion plasmids. Bold letters indicate overlaps and restriction sites are written in emphasized letters.

**Deletion target**	**D1**	**D2**	**D3**	**D4**	**Dfw - Control forward**	**Drv - Control reverse**
cg0016-cg0043 [c]	GCTACAACGACTTCCCATAC	**CCCATCCACCCCGGGTAAACA**TACTTCTCGGGATGCCATAA	**TGTTTACCCGGGGTGGATGGG**CAGCGCAAAGCCACTGAATC	GTCTGCCGCATCAGAAATAG	CAAGGCATTCGAACTTCC	GAGCTGTGCGTTTGTATC
cg0116-cg0147 [c]	AAACGATTGCAGCCGAAG	**CCCATCCACCCCGGGTAAACA**GATCGCAGTGATAATCATGG	**TGTTTACCCGGGGTGGATGGG**GCGAAGCTGCGTGCTGCAAC	ACGCCTCGTTGGTTAAAG	CATACACCCGAAACACTG	CAGGTAGCAACCACAAAG
cg0158-cg0183 [c]	GAAGCCTGCAGTTCAGAAG	**CCCATCCACCCCGGGTAAACA**TGGAAATACGAGAAACCAAAAATTAC	**TGTTTACCCGGGGTGGATGGG**GGGGTTGTAGGAATCGGTGGCAG	GCTGCCTGGCTCAATAAC	GCCCAAAGTATCCAAACC	GTAGCGGAAACTATCGTC
cg0216-cg0232 [b]	GGATCCGCACAGGGTACTGTGAAATGG	**GGAATGGAGTATGGAAGTTGG**AGGATGAACCCGCCTAGTC	**CCAACTTCCATACTCCATTCC**GTTGAATTGCCGCTGCTCC	GAATTCTCTGGTCCAAGAGCACCTCATC	GCTGTTGCGAACTCGATAC	CTGGTCACGTCTCCTATTC
cg0311-cg0333 [b]	GTCGACCAGTGGATCAACGCTGAAGG	**GGAATGGAGTATGGAAGTTGG**CGGTTAGGGCTGTAGGAAGG	**CCAACTTCCATACTCCATTCC**CTACCAAGCTGCGCCATATC	AAGCTTGAGCTGCTTTCATCGAACAC	CAGAAGCGTCTCAACAAG	CGGAACCAACGATTACTG
cg0414-cg0440 [a]	**TAGCTCACTCATTAGGCACC**CTACGTCTTTCCCTCGTGAAC	**GTCATTGTCGTGCATCCGGG**AGAGACATAGCAGGCGTTAC	CCCGGATGCACGACAATGAC	**GCTTTCTACGTGTTCCGCTT**AGAAGTCAACGCCACGGAGG	CTATCGGAGATGGTGGATAG	TTTGGCGCAACAGGGTTAAG
cg0635-cg0646 [a]	**CACTCATTAGGCACC**AGCATCCAAGGGTAAGAC	ATGCCACTCTTGTTACCC	**TAACAAGAGTGGCAT**GGGAGGAGCTTTTCGCAC	**CTACGTGTTCCGCTT**GCCTTCCTTCTTCAACTC	ATCGTCGTCGAAGAAGTG	ACGAATACCTGCGCCAAG
cg0704-cg0748 [c]	GGTACCGCAAACATCAAGAG	**CCCATCCACCCCGGGTAAACA**TTCTAGGATTTCGCTCATCG	**TGTTTACCCGGGGTGGATGGG**CATCTCGCACCACGTATTGCT	CCGCGGTATAAGTTTCCATC	ACCGAACCAGAAGCAAAG	CTTACGGATCGAGTTCAC
cg0822-cg0845 [a]	**GACTCTAGAGGATCCCC**CGCGCATTGAAGGATCTC	**CCCATCCACCCCGGGTAAACA**AAAATATGCAGTTGACATGCG	**TGTTTACCCGGGGTGGATGGG**CGCACCTGATTCAGACATAG	**CGAGCTCGGTACCC**CCATCCCGGTTACCTCTATC	GCTCAACTACGCAAAGAC	CTCACCAACCCTGCAAAG
cg0900-cg0909 [a]	**CACTCATTAGGCACC**AAACCTTGGCGGTAGTGG	CCCCCAACCGAAGTTGAG	**AACTTCGGTTGGGGG**AGACGTCGGTCAAATAGG	**CTACGTGTTCCGCTT**TGCTCGACAACGGCAAAC	GAACCTCTAGCGAATCTC	AATTCGGTGGCGACGTAG

rrnB-cg0931 [a]	**CACTCATTAGGCACC**CGCTACATCGACTTCTTC	AAATCCGCAGGTTGAAGC	**TCAACCTGCGGATTT**AGACGCTCCGCGAAGCTG	**CTACGTGTTCCGCTT**GCCATCGTCATGGTCTTC	CTGGCCAAGATCATCTCC	AGCGACTCCAGATCTCAG
cg1018-cg1033 [a]	**TCACTCATTAGGCACC**CGTTGTCGGTGAGGCCAACGC	**GCATTCACA**CGCCCCACCCCCGCCAAC	**GTGGGGCG**TGTGAATGCAAGGTCAGG	**TCTACGTGTTCCGCTT**CGCAGCAAACACATTGCC	GCGCACTGGAAGAATCTGAC	AGCGCCTCCTCTTGCACATC
cg1172-cg1213 [a]	**TAATGTGAGTTAGCTCACTCATTAGGCACC**TCCACCTGATGACAGCAAAG	**AGTCAGAGGTTTACGTC**AGACGTTAAACCGGAACTCCAC	**GTTCCGGTTTAACGTCTGA**CGTAAACCTCTGACTAGCGTCACCC	**CTGCGGACTGGCTTTCTACGTGTTCCGCTT**CGCCTAAAGGGATTGGAGTG	ACCGCACCTTGTTCTCTG	CAGCCGATAGCTGAGTTG
cg1219-cg1247 [a]	**CACTCATTAGGCACC**TGCGAAGTCGTTCTAGTG	CTCCCGACATATTAGACC	**CTAATATGTCGGGAG**TCGATGCAGTTCCGTTAC	**CTACGTGTTCCGCTT**TGATGACTGGCATCTTCG	CGGCATCGAGTTTGTGTC	CTGTGCTGCTTCTTCATC
cg1281-cg1289 [a]	**CACTCATTAGGCACC**TCTGGCCGAAAGTACTAG	ACCATCAAGTCGGTGAAC	**CACCGACTTGATGGT**TATGTGCCGCTCGGATTG	**CTACGTGTTCCGCTT**ACAGCCATCAACGCCATC	CTGCGCCTCAAAGAGTTC	CGCAGAGCAATCACAGAG
cg1291-cg1305 [a]	**CACTCATTAGGCACC**ATCCGATGCAGTGTTGAC	ACCTTTACGCCACTTCCC	**AAGTGGCGTAAAGGT**AATTGACGCGTGAATGGG	**CTACGTGTTCCGCTT**GTCAAGAACACGCTCTGG	AATAGCGGCGGTATCGAG	GAAAGCTGCAGTCTTACC
cg1308-cg1330 [a]	**CACTCATTAGGCACC**AGAACCTGGCAACCTACC	GTTGCCCTCTCAGTAAAG	**TACTGAGAGGGCAAC**AGTCGACGGTTAGCAATC	**CTACGTGTTCCGCTT**GGCACCAACCAAGCAATC	CCTGGTCAAGGAATACTC	AACCAGCGACCCACAAAC
cg1340-cg1352 [a]	**CACTCATTAGGCACC**AATGCTGCGGCTTCTGTG	TTGTTGGGCCTAAGGTTG	**CCTTAGGCCCAACAA**GAGCTGCGGAATAAAGCC	**CTACGTGTTCCGCTT**GACCATGACGTTGAAGAG	CTGCAGCTAATGGTTTGG	AACGAGGGCGAGATTCAG
cg1370-cg1385 [a]	**CACTCATTAGGCACC**GTCGAAGCTGCATACAAG	GCGACACCCAGAAATACC	**ATTTCTGGGTGTCGC**CGCGCCTAAAGCCGATGC	**CTACGTGTTCCGCTT**ACTGAGCTGGATTGCTTC	CGCTTTCGAGCGTATTTG	AACCCGTCACGATGAGAG
cg1540-cg1549 [a]	**CACTCATTAGGCACC**AACCGGCATTAGCAGAGC	CGAGGGCTTTCTTGGTAG	**CCAAGAAAGCCCTCG**TTTGCGCTAAACGTAGGG	**CTACGTGTTCCGCTT**CGCCTACTCAGCAAAGAC	GCGGTAAATCTACCGTTG	CACAACGGAACGGTCAAG
cg1843-cg1853 [a]	**CACTCATTAGGCACC**GTCCGTCAGACCAAGATG	CCGGAATTAATCGGTGTG	**ACCGATTAATTCCGG**CCCACATCGTGAATTGAC	**CTACGTGTTCCGCTT**GCATGGTTCGTGGTTTGG	TACACCGAGCCGACATTG	TGCTGGATCACCTTGATG
cg2136-cg2139 [a]	**CACTCATTAGGCACC**TTCCGCTTGGGCATTGTG	GGAGACGAGCCTTGTTAG	**ACAAGGCTCGTCTCC**CCTTACCCCGGATGATTC	**CTACGTGTTCCGCTT**AAGCCAACGTACGCAGAC	ACACCCAACCAGCGTAAG	CTTTGAACGCGCTCATGC

cg2312-cg2322 [a]	**GACTCTAGAGGATCCCC**CTGGAGGTCGTGAAACAAC	**CCCATCCACCCCGGGTAAACA**GCGCTGCAGGCGTTGAAGTC	**TGTTTACCCGGGGTGGATGGG**CCTTACCAGCCAGCCTTTGTAG	**CGAGCTCGGTACCC**GGCGCTTGTAGACTTCTTG	CGGACGAAGAAGAGAAGTAG	GGTTCATCAACGCTCAAG
cg2348-cg2358 [a]	**CACTCATTAGGCACC**GAACAACCGGTCCAACAC	TGGCCGAATTTTTGCTGC	**GCAAAAATTCGGCCA**GCAGCGGGCTTTCTCAAC	**CTACGTGTTCCGCTT**GAACCTTCGAGGTTGTTG	ACGCCGCAAGAAATCGAG	ACTTCGCTTCGATCATCC
cg2539-cg2560 [b]	GGATCCTGGGAAAGCCACGATGATAACC	**GGAATGGAGTATGGAAGTTGG**CTGGCAGGGCAAATGGAC	**CCAACTTCCATACTCCATTCC**GGACGTGACTGAACATGAC	GAATTCATCCCAACGGTGACTGAC	GAACCGCAGCAGCATCTTC	GCTACACCGAGCCCATCTTG
cg2561-cg2570 [b]	GGATCCCTTGAGCTGCACTAATTCAC	**GGAATGGAGTATGGAAGTTGG**CGACGATCCAGATGAACAC	**CCAACTTCCATACTCCATTCC**CGGCATCAGTTTCTGTGTTCC	GAATTCGCACCAAATGAGCGTGATCC	AGGTCTTCGGCGATCATAC	TTGTCGTGCTTCGGTGTG
cg2621-cg2643 [a]	**GCAACGCAATTAATGTGAGTTAGCTCACTCATTAGGCACC**CGCGGAGACGCTTGTAATGG	AGTTGGCGTGGAAGATGTGG	**CCACATCTTCCACGCCAACT**TAAGGAACCAAACCCGAATC	**TCTACGTGTTCCGCTT**AGGCCCAATCAGCAACTAGG	ATAACCAGCCTCGGTCAG	ACACCGGCCAGACCTTTGAG
cg2663-cg2686 [a]	**TCACTCATTAGGCACC**CGAGGCTGTCGAAGATGC	**CGGCAAGC**TTTGTGTTTTGTTCATTGTTTTCCC	**AAAACACAAA**GCTTGCCGCCAAATAGTTTC	**TCTACGTGTTCCGCTT**ACTCCGACGTTGTTGGCG	GCTGCTGCCGATTACTTCCG	ATACCTCCGTCGTGGAAG
cg2701-cg2716 [b]	GGATCCCGAATCGGTAGCCGCTTCTG	**GGAATGGAGTATGGAAGTTGG**GCGGCTCGGCCTTCGTTCG	**CCAACTTCCATACTCCATTCC**TGGCATCGCGGACGTAAC	AAGCTTGCTCTTGCTGGGTAGTGGTAAC	GTGGGTGATCCTTGCCTTTC	GATCTGCGGCGATCTCTTTG
cg2755-cg2760 [a]	**TCACTCATTAGGCACC**GAACATCGCAGTCAATCGC	**GGCTATTTTT**TGGGCTTTCCGCACTGTT	**AAAGCCCA**AAAAATAGCCCCTTCCGGG	**TCTACGTGTTCCGCTT**TTGCTTTCGACGCCTGGG	GCTCCTGCAGAACCTTGATG	TGATGGTGTGGCGCAGGTTC
cg2801-cg2828 [a]	**TCACTCATTAGGCACC**GATGGGGTGATCACGATG	**AGGGGCTG**TACTCCGTGAATTTTGCC	**ACGGAGTA**CAGCCCCTCTGACAAGCA	**TCTACGTGTTCCGCTT**GCACCTTCCATTGGAAAAGC	ATATCGTTGGCGCGGTTGGC	AAGGCGTAGAGAAACTCACC
cg2880-cg2904 [a]	**TCACTCATTAGGCACC**GGCCGAAACGATCGATGG	**TCCCGTTT**TTGCATAAAATAATGCAGGTGAGC	**TTTTATGCAA**AAACGGGAGCCTAAGTGAAATG	**TCTACGTGTTCCGCTT**TCGCAGCCAAATTCATCAC	CCCTCAAGCACTGCATCTAC	ACGTGCGTGTAGTACAAACC
cg2925-cg2943 [b]	GGATCCTGTGGATGCTCTCCTTTCTG	**GGAATGGAGTATGGAAGTTGG**CACTAATGCGGTGGAGTTG	**CCAACTTCCATACTCCATTCC**GGCGCAGCGATTGCTTATGGC	GTCGACTGTTGCTGGCACCGCTTCTTTC	CTCCGACGGTGATGTTGTAG	GGGTACCGGTGTCAAAGATG
cg2965-cg2973 [b]	GTCGACCCGACGGACGCATGTACAAAG	**GGAATGGAGTATGGAAGTTGG**ATTGATGGGAGGTGCAGGTTGG	**CCAACTTCCATACTCCATTCC**CGCAGAAGTCTCGGTATCG	CTGCAGTCACCGCAATGGAACAAGG	ATGGTCGGATCCTGGTTCTC	GAACTAGCAACCGGATACTC

cg2990-<br>cg3006 [b]	CCGGAATTCAACCGTCGAAATC<br>CCTAAGGG	**CCCATCCACTAAACTTAAACA**<br>AATCGCAGGGATGCGCAGC	**TGTTTAAGTTTAGTGGATGGG**<br>TACTGAAAGGTGCTGGGGAG	CGCGGATCCTTGACCGCTCTTG<br>GTACAACC	ACACGCTA<br>GAATTCAA<br>GGGCAG	GTACTTCCA<br>GCGCTATA<br>TTCAGC
cg3050-<br>cg3062 [a]	**TCACTCATTAGGCACC**AAGAC<br>CTGGGATATGCAGTC	**TGGCTCCG**GGACGAGCATTCA<br>CCGGA	**GCTCGTCC**CGGAGCCATTAAT<br>GGACTTG	**TCTACGTGTTCCGCTT**AAGTG<br>GGGCGAGTACCTG	GAGGACGG<br>CAAGGTTG<br>TTGG	TCTCCTACG<br>CTGATCGTC<br>TG
cg3072-<br>cg3091 [b]	CCGGAATTCAGCACCTTCTTGC<br>CCGTC	**CCCATCCACTAAACTTAAACA**<br>ACGCGGATGAGCCACCAA	**TGTTTAAGTTTAGTGGATGGG**<br>GCTTCCAATCCCGCATTGTTC	CGCGGATCCGCCGCTTCTTTAA<br>AGAACTCCAG	GCTGTCAG<br>AGGGGAAT<br>TTCC	ACCCAAAG<br>CGCTGTGC<br>ATTTC
cg3102-<br>cg3111 [b]	CCGGAATTCCTGTCATCATCGC<br>GCTAGTTAC	**CCCATCCACTAAACTTAAACA**<br>AGTTGGCGGAGGCTGTGAAG	**TGTTTAAGTTTAGTGGATGGG**<br>GCCCAACAAGCCGTGTCGTT	CGCGGATCCCAACGAGACCAT<br>CTCCAGC	CGGTCATC<br>ACATTCTTC<br>GCG	CTGCCGCT<br>GACGTACC<br>TC
cg3119-<br>cg3173 [a]	**GACTCTAGAGGATCCCC**CATC<br>CTGCAGCTCATCATC	**CCCATCCACCCCGGGTAAAC**<br>**A**GCGCAGGGGAGTTGTCATGT	**TGTTTACCCGGGGTGGATGG**<br>**G**GCCTTGTTATCCAACCGCCAC<br>AATTC	**CGAGCTCGGTACCC**CCAGAA<br>GGGTCACAACAG	GATCGCAG<br>GCACATCTT<br>C	GGGTGCCC<br>TTATCCATT<br>TC
cg3208-<br>cg3236 [b]	CCGGAATTCGACCATGATGGA<br>AAAGGCGATG	**CCCATCCACTAAACTTAAATC**<br>**A**GGTGGGCATGCCTGCGAC	**TGATTTAAGTTTAGTGGATGG**<br>**G**CTTATGCCCTTCAACCCTACT<br>TAG	CGCGGATCCAGTTCGCCGGTAG<br>GCTCG	GGTTTTGAC<br>GTCGGCAA<br>GC	CTTAGCGA<br>AAGAACCG<br>AAGTCG
rrnC-<br>cg3298 [a]	**CACTCATTAGGCACC**TTGTTT<br>GCCCGACGTGAG	AAGTGCTCGAAGCGACAG	**TCGCTTCGAGCACTT**GCCGAG<br>AAGAAGTTCTCC	**CTACGTGTTCCGCTT**CGATGT<br>GCAGTGAATCAG	CGTGTGCT<br>GACTGGAA<br>AC	CTTCCTTCC<br>GATGTGAT<br>G
cg3263-<br>cg3301 [a]	**GACTCTAGAGGATCCCC**GCCT<br>CACTGTCCACATAATC	**CCCATCCACCCCGGGTAAAC**<br>**A**CTGTGTGCGTTCAAGCATGG	**TGTTTACCCGGGGTGGATGG**<br>**G**CTGCCTGAGCAGAAGGGTAA	**CGAGCTCGGTACCC**GGATCCG<br>GAGAATTCAGG	CTTGTTGGC<br>AGCTTGTTC	GGCAAGAC<br>TTACCGAC<br>ATAG
cg3324-<br>cg3345 [b]	CCGGAATTCGGCGGCAACATG<br>GACTTC	**CCCATCCACTAAACTTAAACA**<br>AACGTGAATCTGGCAAAAGAA<br>TTGGG	**TGTTTAAGTTTAGTGGATGGG**<br>GAAAACCTCGCCTGCCCACT	CGCGGATCCCGTCCGCGACGAC<br>TACAC	AACGTGGC<br>GTTCGCGT<br>AG	ACCGACGA<br>TGACAACA<br>AGCAAC
cg3365-<br>cg3413 [a]	**GACTCTAGAGGATCCCC**CATC<br>GGAATGCTCATCTACG	**CCCATCCACCCCGGGTAAAC**<br>**A**CCGGCGGGCGCTCCTACCCCA<br>C	**TGTTTACCCGGGGTGGATGG**<br>**G**TAAATTTTCGGAACTCATCTA<br>ATTATC	**CGAGCTCGGTACCC**GGACCTG<br>AAGTGGGTAAG	CAGATGCA<br>CTGGAACT<br>TG	GCCACCTA<br>CGATCATTT<br>G
